# Evaluation of liposome**-**encapsulated *Centella asiatica* ethanolic extract for enhanced *in vitro* and *in vivo* wound healing

**DOI:** 10.3389/fmedt.2026.1740835

**Published:** 2026-01-23

**Authors:** Piriya Chonsut, Weeratian Tawanwongsri, Jomkarn Naphatthalung, Julalak Chokpaisarn, Rawiwan Charoensup, Panupong Puttarak, Siau Hui Mah, Atitaya Roumwong, Lavanya Goodla, Auemphon Mordmuang

**Affiliations:** 1 Department of Applied Thai Traditional Medicine, School of Medicine, Walailak University, Nakhon Si Thammarat, Thailand; 2 Center of Excellence in Tropical Pathobiology, Walailak University, Nakhon Si Thammarat, Thailand; 3 Department of Dermatology, School of Medicine, Walailak University, Nakhon Si Thammarat, Thailand; 4 Traditional Thai Medical Research and Innovation Center, Faculty of Traditional Thai Medicine, Prince of Songkla University, Songkhla, Thailand; 5 School of Integrative Medicine, Mae Fah Luang University, Chiang Rai, Thailand; 6 Medical Plants Innovation Center, Mae Fah Luang University, Chiang Rai, Thailand; 7 Department of Pharmacognosy and Pharmaceutical Botany, Faculty of Pharmaceutical Sciences, Prince of Songkla University, Hat Yai, Songkhla, Thailand; 8 Phytomedicine and Pharmaceutical Biotechnology Excellence Center, Faculty of Pharmaceutical Sciences, Prince of Songkla University, Hat Yai, Songkhla, Thailand; 9 School of Biosciences, Faculty of Health and Medical Sciences, Taylor's University, Subang Jaya, Malaysia; 10 Department of Anatomy, Faculty of Medicine, Chulalongkorn University, Bangkok, Thailand; 11 Department of Biochemistry and Molecular Biology, University of New Mexico School of Medicine, Albuquerque, NM, United States; 12 Department of Medical Sciences, School of Medicine, Walailak University, Nakhon Si Thammarat, Thailand

**Keywords:** anti-inflammatory, *Centella asiatica*, drug delivery, liposome, proliferative activity, wound healing

## Abstract

**Introduction:**

Encapsulating herbal extracts with wound-healing properties in liposomes may enhance their stability and delivery performance. This study evaluated the biological efficacy of a liposome-encapsulated ethanolic extract of *Centella asiatica* (LEC) using in vitro and *in vivo* wound-healing models.

**Methods:**

The ethanolic extract was incorporated into liposomes using the thin-film hydration method. Anti-inflammatory activity was assessed in lipopolysaccharide (LPS)-stimulated RAW 264.7 macrophages. Cell viability and migration were evaluated in normal human dermal fibroblasts (NHDFs). *In vivo* wound-healing efficacy was examined using a rat excision wound model with daily topical application of LEC.

**Results:**

LEC significantly reduced TNF-α and IL-1β production in a dose-dependent manner and enhanced fibroblast viability and migratory capacity compared with the crude extract and vitamin E controls. In vivo, topical LEC markedly accelerated wound contraction, achieving 99.9 ± 0.1% closure by Day 12, which was significantly greater than the normal saline-treated control (*p* < 0.05) and higher than the blank liposome group, while demonstrating comparable efficacy to vitamin E. Histological analysis revealed enhanced re-epithelialization, increased collagen deposition, and reduced inflammatory cell infiltration in LEC-treated wounds.

**Conclusion:**

These findings indicate that liposomal encapsulation enhances the bioactivity of *C. asiatica* extract during the inflammatory and proliferative phases of wound repair, supporting further development of LEC as a topical wound-healing formulation.

## 1 Introduction

The healing of chronic or acute wounds is a highly coordinated biological process that involves inflammation, tissue formation, and tissue remodeling. This process is regulated by a variety of molecular signals, including growth factors, cytokines, and extracellular matrix components, as well as cellular events such as cell migration, proliferation, and differentiation (1, 2). While many pharmaceutical treatments have been developed to expedite wound healing, there is still a significant demand for effective, natural, and safe therapies, especially for chronic wounds, which often fail to heal with conventional treatments.

*Centella asiatica* (L.) Urb. [Apiaceae], also known as Gotu Kola, is a medicinal plant widely used in traditional medicine for promoting wound healing and skin regeneration (3–6). Its bioactive compounds, including asiaticoside, madecassoside, and centelloside (6, 7), have been shown to stimulate collagen synthesis, enhance fibroblast proliferation, and accelerate re-epithelialization in skin wounds. However, the clinical efficacy of *C. asiatica* is limited by its low bioavailability when administered orally or topically due to its poor absorption and rapid degradation *in vivo*. Liposome encapsulation has emerged as an effective strategy for improving the bioavailability and stability of plant-derived active compounds. Liposomes are lipid-based vesicles that can encapsulate both hydrophilic and lipophilic substances, protecting them from degradation and facilitating their absorption through biological membranes (8, 9).

The ability of the liposomal formulation to reduce inflammation, promote fibroblast viability, and enhance cell migration, are essential component of the wound healing process (10, 11). The inflammatory phase is critical in wound healing, involving the recruitment of immune cells and the release of pro-inflammatory cytokines like TNF-α and IL-1β (12, 13). While necessary for initiating tissue repair, excessive inflammation can impede wound healing and lead to chronic wounds (10). Reducing the levels of these cytokines helps mitigate prolonged inflammation, creating a conducive environment for tissue regeneration (1, 14). The *in vitro* model such as cellular proliferation and survival is essential for tissue formation during wound healing. Fibroblasts, a key cell type in this process, play a pivotal role in synthesizing extracellular matrix components, including collagen, which provides structural integrity to the forming tissue (11, 15, 16). Moreover, the evaluation of cell migration, particularly of fibroblasts, is vital for wound closure and re-epithelialization (2, 15). To assess these mechanisms, we employed both *in vitro* and *in vivo* models. The *in vitro* studies focused on evaluating anti-inflammatory effects, cell viability, and fibroblast migration, using established assays. To validate these findings in a physiological context, we further conducted an *in vivo* wound healing experiment using healthy male Wistar albino rats. A full-thickness excision wound model was established, and rats received daily topical applications of the test agents. Wound contraction was measured at defined intervals,

Together, these approaches enabled a comprehensive evaluation of the liposome-encapsulated *C. asiatica* extract. This study aims to determine its potential to promote wound healing through both anti-inflammatory and proliferative mechanisms. The findings provide valuable insights into the therapeutic applications of this formulation for effective and accelerated wound care. To further validate the *in vitro* findings, we incorporated an *in vivo* wound healing model using healthy male Wistar albino rats. A full-thickness excision wound model was employed, in which rats received topical applications of all agents. Wound contraction was measured periodically, and the percentage of wound closure was calculated to assess healing efficacy.

## 2 Materials and methods

### 2.1 Plant and extraction

Fresh *C. asiatica* leaves were sourced from Chian Yai Subdistrict and Mae Chao Yu Hua Subdistrict, Nakhon Si Thammarat Province, Thailand. Herbarium voucher specimens were *C. asiatica* SMD 0324030901. They were deposited at Applied Thai Traditional Medicine, School of Medicine, Walailak University, Nakhon Si Thammarat, Thailand. The plant materials were dried using a hot air oven at 60 °C for 72 h. All dried herbs were ground into coarse powders. To obtain crude ethanolic extracts, pulverized herbs were macerated in 95% ethanol (1:10 w/v) for 7 days with occasional stirring. The extract was filtered and concentrated under reduced pressure using a rotary evaporator. The final ethanolic extract was stored at 4 °C for further use.

### 2.2 Preparation and characterization of liposome-encapsulated *Centella asiatica* ethanolic extract (LEC)

Liposomes were prepared using the thin-film hydration method. Briefly, phosphatidylcholine (30 mg) and cholesterol (10 mg) were dissolved in chloroform (10 mL) in a round-bottom flask, followed by the addition of the ethanolic extract of *Centella asiatica* (10 mg). The solvent mixture was evaporated under reduced pressure using a rotary evaporator at 40 °C and 150 rpm to form a uniform thin lipid film along the inner wall of the flask. The resulting lipid film was hydrated with 5 mL of phosphate-buffered saline (PBS, pH 7.4) and vortexed for 5 min to obtain a milky liposomal suspension.

The liposomal dispersion was subsequently extruded through a 100 nm polycarbonate membrane using a mini-extruder (Avanti Polar Lipids, USA) to obtain vesicles with a uniform size distribution. Unencapsulated extract was removed by centrifugation at 15,000 × g for 30 min at 4 °C, and the resulting liposomal pellet was re-dispersed in PBS and stored at 4 °C until further use.

The UV–visible absorption spectra (200–800 nm) of both the ethanolic extract and the liposomal formulation were recorded to identify characteristic absorption peaks of phytoconstituents and to quantify extract content using pre-established calibration curves of reference standards.

The hydrodynamic particle size and polydispersity index (PDI) of the liposomes were determined by dynamic light scattering (DLS) using a Zetasizer Nano ZS (Malvern Panalytical, Malvern, UK) at 25 °C. Measurements were performed in triplicate, and representative size distribution profiles are presented.

The zeta potential of the liposomal formulation was measured by electrophoretic light scattering using the same Zetasizer Nano ZS instrument. Samples were appropriately diluted with deionized water to avoid multiple scattering effects and analyzed at 25 °C. Zeta potential values were calculated from electrophoretic mobility using the Smoluchowski approximation. Measurements were conducted in triplicate, and results are reported as the observed measurement range (−35 to −25 mV), indicating moderate colloidal stability of the liposomal formulation (36).

Encapsulation efficiency (EE%) of the liposome-encapsulated *C. asiatica* ethanolic extract was determined by quantifying the amount of extract entrapped within the liposomal vesicles. Briefly, the liposomal dispersion was centrifuged at 15,000 × g for 30 min at 4 °C to separate unencapsulated (free) extract from the liposome-associated fraction. The supernatant containing the free extract was carefully collected, and its absorbance was measured using a UV–visible spectrophotometer at the characteristic wavelength of the extract, based on previously established calibration curves.

$$\begin{array}{rl} & \mathrm{Encapsulation}\;\mathrm{efficiency}\;\mathrm{was}\;\mathrm{calculated}\;\mathrm{using}\;\mathrm{the}\;\mathrm{following}\;\mathrm{equation}:\\ & \;\;\;\mathrm{EE}(\text{\%})=(\mathrm{Total}\;\mathrm{extract}-\mathrm{Free}\;\mathrm{extract}/\mathrm{Total}\;\mathrm{extract})\times 100 \end{array}$$

### 2.3 Cell culture

Mouse macrophage cells (RAW 264.7, ATCC TIB-71) were used for anti-inflammatory assays, while normal human dermal fibroblasts (NHDF; ATCC PCS-201-012™) were employed to model skin wound healing. Both cell lines were obtained from the Faculty of Medicine, Chulalongkorn University (Bangkok, Thailand). Cells were cultured in Dulbecco's Modified Eagle Medium (DMEM) supplemented with 10% fetal bovine serum (FBS) and 1% penicillin-streptomycin and maintained at 37 °C in a humidified incubator with 5% CO₂. The study included four treatment groups: Negative control (Blank Liposome): Liposomes prepared without *C. asiatica* extract. Positive Control (Vitamin E). LEC Group (Liposome-Encapsulated Extract): Liposome-encapsulated *C. asiatica* ethanolic extract. EE Group (Ethanolic Extract): Crude ethanolic extract of *C. asiatica*.

### 2.4 *In vitro* anti-inflammation on LPS-induced RAW 264.7 cells

RAW 264.7 cells were pre-treated with LPS (1 μg/mL) for 24 h to induce an inflammatory response. Cells were then treated with different concentrations of liposome-encapsulated *C. asiatica* ethanolic extract, blank liposome base, ethanolic extract, or vitamin E. The levels of pro-inflammatory cytokines (TNF-α, IL-1β) in the culture supernatants were measured by enzyme-linked immunosorbent assay (ELISA, BD Biosciences, San Jose, CA) after 24 h of treatment (17, 18).

### 2.5 Modeling skin wound healing

#### 2.5.1 Cell viability by MTT assay

The viability of Normal human dermal fibroblasts (NHDF) cells was evaluated following 24 h exposure to liposome-encapsulated *C. asiatica* extract, crude ethanolic extract, or respective controls at concentrations ranging from 0 to 100 µg/mL. NHDF cells were seeded at a density of 1 × 10^4^ cells per well in 96-well plates and treated with increasing concentrations of each test substance for 24 h. Cell metabolic activity was then assessed using the MTT assay, and absorbance was measured at 570 nm to determine cell viability (19).

#### 2.5.2 Cell migration by scratch assay

NHDF cells were grown to confluence in 24-well plates. A linear scratch was created using a sterile pipette tip, and the cells were treated with various concentrations of liposome-encapsulated *C. asiatica* extract, the ethanolic extract, or controls. Wound closure was monitored at 0, 12, and 24 h using a light microscope. The percentage of wound closure was calculated by measuring the gap at each time point (20, 21).

### 2.6 Wound healing in a mouse model

Healthy male Wistar albino rats (150–200 g) were housed in standard stainless-steel cages under controlled environmental conditions (12 h light/dark cycle, 21 ± 1 °C, 50 ± 10% relative humidity) with free access to food and water. After a 7-day acclimatization period, twenty animals were randomly assigned to experimental groups. All animal procedures complied with institutional and national ethical guidelines and were approved by the Institutional Animal Care and Use Committee (IACUC), Prince of Songkla University, Thailand (Ethics Approval No. AR029/2025).

#### 2.6.1 Excision wound model

Rats were anesthetized with thiopental sodium (60 mg/kg, intraperitoneally). The dorsal surface was shaved and disinfected with 70% ethanol. Two full-thickness excision wounds (8 mm in diameter and approximately 2 mm deep) were created on the dorsum of each rat under aseptic conditions using a sterile 8 mm biopsy punch, as previously described (22, 23). Hemostasis was achieved by gentle blotting with sterile gauze. The animals were randomly divided into four groups (*n* = 5 per group) as follows: Normal saline control: wounds treated with normal saline solution; Blank liposome control: wounds treated with the liposomal gel base without extract; LEC group: wounds treated with liposome-encapsulated *C.asiatica* extract gel (10 mg/g; 1% w/w) applied at a dose of 10 mg/cm² wound area; and vitamin E group: wounds treated with a commercial vitamin E gel (positive control). The assigned treatments were applied topically once daily until complete epithelialization. Wound areas were measured every other day using a digital vernier caliper, and the percentage of wound contraction was calculated using the following formula:

$$\mathrm{Percentage}\;\mathrm{contraction}=\frac{\mathrm{Initial}\;\mathrm{wound}\;\mathrm{area}-\mathrm{Current}\;\mathrm{wound}\;\mathrm{area}}{\mathrm{Initial}\;\mathrm{wound}\;\mathrm{area}}\times 100$$

The time to complete epithelialization was recorded as the number of days required for complete wound closure, defined by the absence of a visible wound gap and scab formation, as assessed by daily macroscopic observation.

#### 2.6.2 Histopathological examinations

Wound tissues were harvested from all experimental groups on the termination day and subjected to histopathological evaluation. Excised tissues were gently rinsed PBS and immediately fixed in 10% neutral buffered formalin for 24 h. Following fixation, samples were dehydrated through a graded ethanol series, cleared in xylene, and embedded in paraffin wax. Paraffin-embedded tissues were sectioned at 4–5 µm thickness using a rotary microtome and mounted onto glass slides.Sections were stained with hematoxylin and eosin (H&E) to evaluate overall tissue architecture, inflammatory cell infiltration, fibroblast proliferation, neovascularization, and re-epithelialization. Masson's trichrome staining was additionally performed to assess collagen deposition and extracellular matrix remodeling (24, 25). Histological images were captured using a light microscope at appropriate magnifications Semi-quantitative histological evaluation of re-epithelialization, inflammatory cell infiltration, fibroblast/granulation tissue formation, and neovascularization was performed using a standardized scoring system. Quantitative analysis of collagen area fraction was conducted using Image J software.

## 3 Statistical analysis

Statistical analyses were performed using GraphPad Prism (version 8.0.2.; GraphPad Software, San Diego, CA, USA). Data are presented as mean ± standard deviation (SD). For *in vitro* experiments involving multiple treatment groups and concentrations (MTT assay, fibroblast migration, and cytokine ELISA), data were analyzed using two-way analysis of variance (ANOVA) with treatment and concentration as independent factors, followed by Tukey's multiple-comparison *post hoc* test. For *in vivo* wound-healing studies, where wound closure was measured repeatedly over time, data were analyzed using two-way repeated-measures ANOVA with treatment and time as factors, followed by Tukey's multiple-comparison test. For histological semi-quantitative scores, statistical comparisons were performed using the Kruskal–Wallis test followed by Dunn's multiple-comparison test, while collagen area fraction (%) quantified from Masson's trichrome staining was analyzed using one-way ANOVA followed by Tukey's *post hoc* test. A value of *p* *<* 0.05 was considered statistically significant.

## 4 Results

### 4.1 Characterization and encapsulation efficiency of LEC

**Figure 1:**
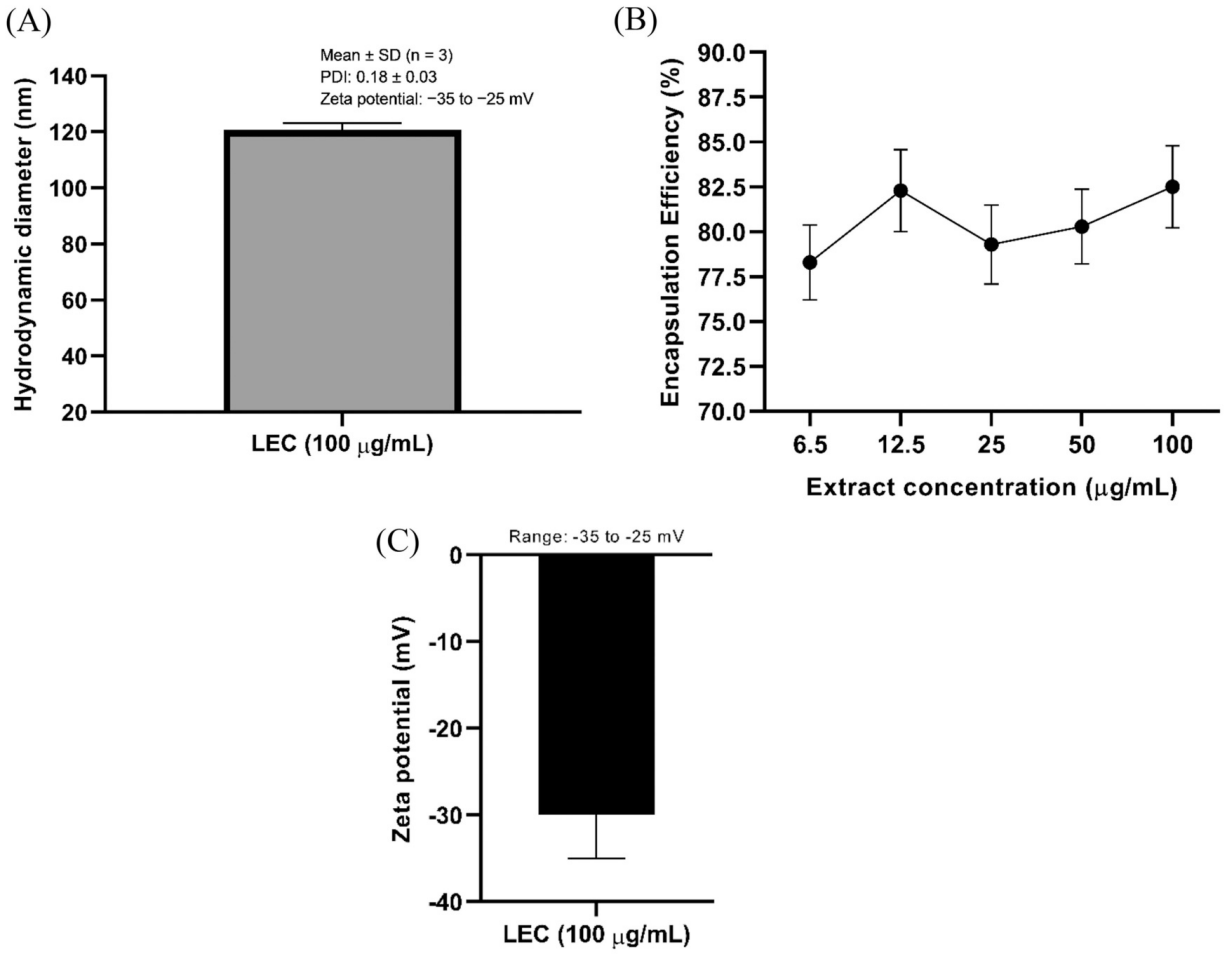
Physicochemical characterization and encapsulation efficiency of liposome-encapsulated *C. asiatica* ethanolic extract (LEC). **(A)** Particle size characterization of the selected LEC formulation (100 µg/mL) determined by dynamic light scattering (DLS), showing mean hydrodynamic diameter ± SD (*n* = 3) and polydispersity index (PDI). **(B)** Concentration-dependent encapsulation efficiency (EE%) of LEC at extract concentrations ranging from 6.5 to 100 µg/mL are presented as mean ± SD from three independent liposome preparations (*n* = 3). **(C)** Zeta potential of the LEC formulation measured by electrophoretic light scattering at 25 °C using the Smoluchowski approximation. Data are presented as the midpoint of the observed measurement range, with error bars indicating the range (−35 to −25 mV).

DLS analysis of the selected LEC formulation (100 µg/mL) demonstrated nanosized vesicles with a mean hydrodynamic diameter of 120.6 ± 9.4 nm and a low polydispersity index (PDI) of 0.18 ± 0.03 (Figure 1A), indicating a narrow size distribution and good formulation homogeneity. The zeta potential of this formulation ranged between −35 and −25 mV, suggesting sufficient surface charge to ensure colloidal stability and minimize vesicle aggregation during storage (Figure 1C). Encapsulation efficiency (EE%) was evaluated across the tested extract concentration range (6.5–100 µg/mL), reaching a maximum EE of 82.5 ± 2.3% (Figure 1B). The high encapsulation efficiency indicates favorable interactions between the bioactive phytoconstituents and the lipid matrix, supporting the suitability of the liposomal system for enhanced delivery and subsequent *in vitro* and *in vivo* applications.

### 4.2 Anti-inflammation effects of the liposome-encapsulated *C. asiatica* extract

**Figure 2:**
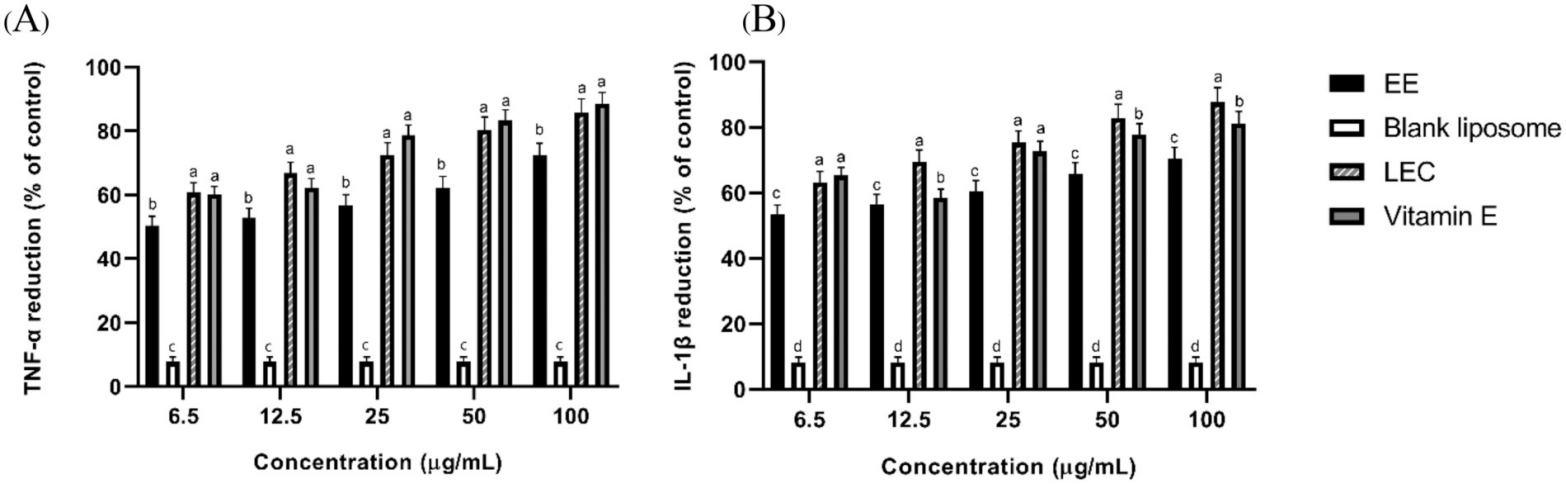
Anti-inflammatory effects of liposome-encapsulated *C. asiatica* extract in LPS-stimulated RAW 264.7 macrophages. **(A)** TNF-α and **(B)** IL-1β production following treatment with EE, blank liposome, LEC, and vitamin E at concentrations of 6.5–100 µg/mL. Cytokine levels were quantified by ELISA and expressed as relative values normalized to the LPS-treated control (set to 100%). Data are presented as mean ± SD (*n* = 3). Statistical analysis was performed using two-way ANOVA followed by Tukey's multiple-comparison test. Different letters above bars indicate statistically significant differences between treatment groups at the same concentration (*p* *<* *0.05*).

The liposome-encapsulated *C. asiatica* extract demonstrated significant inhibition of pro-inflammatory cytokines compared to the blank liposome base. The reduction in TNF-α and IL-1β levels was more pronounced than with the crude ethanolic extract, indicating superior anti-inflammatory efficacy of the liposomal formulation (Figures 2A,B). The results of the study demonstrate that liposome-encapsulated *C. asiatica* extract (LEC) significantly reduced the levels of pro-inflammatory cytokines TNF-α and IL-1β in a dose-dependent manner (*p* < 0.05) compared to both the ethanolic extract (EE) and the blank liposome base. At lower concentrations (6.5–12.5 µg/mL), LEC achieved a substantial reduction in cytokine levels, indicating enhanced anti-inflammatory efficacy even at minimal doses. For example, at 12.5 µg/mL, LEC reduced TNF-α and IL-1β levels by 66.8 ± 3.4% and 69.5 ± 3.5%, respectively (Figures 1, 2), which was significantly greater than the reductions observed in the EE group at the same concentration (52.8 ± 3.0% for TNF-α and 56.4 ± 3.1% for IL-1β; *p* < 0.05). At higher concentrations (50–100 µg/mL), the efficacy of LEC was further amplified, with reductions of 80.4 ± 4.0% and 85.7 ± 4.3% for TNF-α at 50 and 100 µg/mL, respectively. These values were superior to the positive control group (vitamin E), which showed 83.2 ± 3.4% and 88.5 ± 3.5% reductions at the same concentrations. This suggests that LEC could serve as a more effective alternative or complementary anti-inflammatory treatment. The ethanolic extract (EE) showed moderate efficacy but was consistently less effective than LEC across all tested concentrations. For instance, at 100 µg/mL, EE reduced TNF-α levels by 72.5 ± 3.6%, markedly lower than LEC at the same concentration (85.7 ± 4.3%). The blank liposome base exhibited negligible anti-inflammatory activity, with cytokine reductions below 10% in all cases, confirming that the observed effects were primarily due to the active compounds in *C. asiatica* (Figures 2A,B).

### 4.3 Liposome-encapsulated *C. asiatica* extract enhanced cell viability

**Figure 3:**
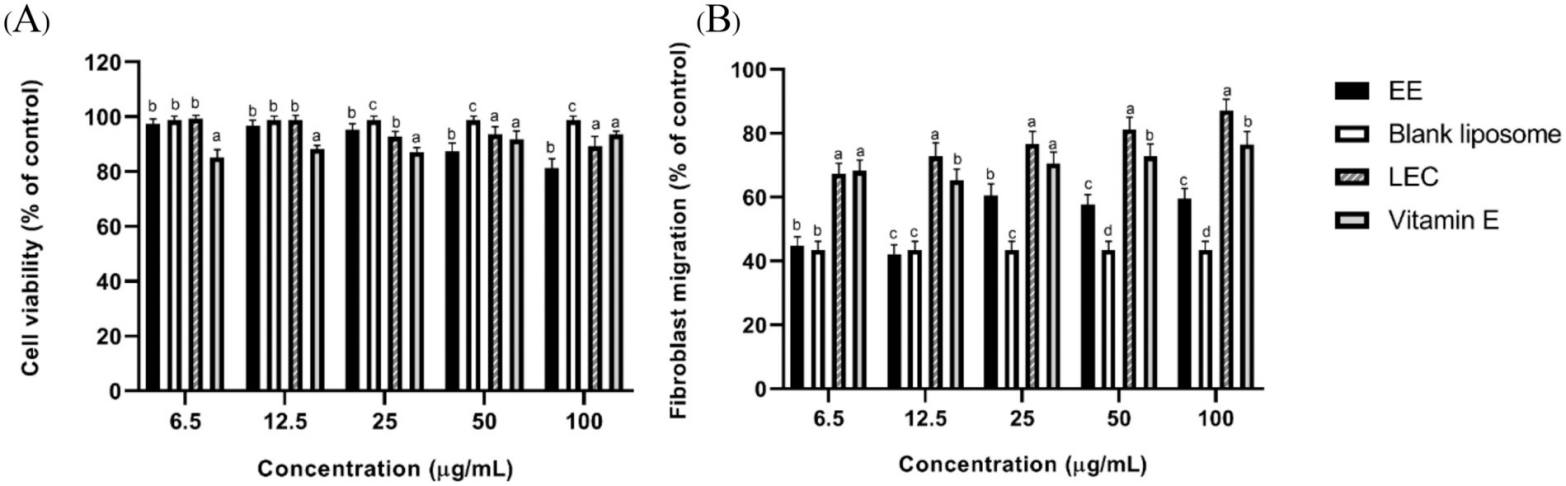
Effects of liposome-encapsulated *C.asiatica* extract on fibroblast viability and migration. **(A)** Cell viability of normal human dermal fibroblasts (NHDFs) treated with EE, blank liposome, LEC, or vitamin E at concentrations of 6.5–100 µg/mL, as determined by the MTT assay. **(B)** Fibroblast migration assessed by scratch-wound assay following treatment with the same formulations and concentrations. Results are expressed as relative values normalized to the untreated control (set to 100%) and presented as mean ± SD (*n* = 3). Statistical analysis was performed using two-way ANOVA followed by Tukey's multiple-comparison test. Different letters above bars indicate statistically significant differences between treatment groups at the same concentration (*p* *<* *0.05*).

LEC exhibited superior cell viability across all tested concentrations, demonstrating minimal cytotoxicity compared to the ethanolic extract (EE) and positive control (vitamin E). At the lowest concentration (6.5 µg/mL), LEC maintained cell viability at 99.2 ± 1.4%, slightly higher than EE (97.4 ± 1.9%) and significantly better than vitamin E (85.3 ± 2.8%). Even at the highest concentration (100 µg/mL), LEC displayed a viability of 89.2 ± 3.6%, indicating its safety and biocompatibility for wound healing applications. In contrast, EE showed a progressive decline in cell viability at higher concentrations, with a viability of 81.2 ± 3.5% at 100 µg/mL. This decline suggests potential cytotoxic effects at higher doses. Vitamin E exhibited consistent viability (85%–93%) across all concentrations but was slightly less effective than LEC, particularly at lower concentrations. The blank liposome control exhibited high viability (98.7 ± 1.5%), confirming that the liposome base itself is non-toxic (Figure 3A).

### 4.4 Liposome-encapsulated *C. asiatica* extract promoted cell migration

Fibroblast migration, a critical factor for wound healing, was significantly enhanced by LEC, particularly at concentrations between 12.5–50 µg/mL. At 12.5 µg/mL, LEC achieved a migration rate of 72.8 ± 4.2%, surpassing both EE (42.2 ± 2.8%) and vitamin E (65.3 ± 3.5%) at the same concentration. The highest migration rate for LEC was observed at 100 µg/mL (87.2 ± 3.7%), which outperformed all other groups, including vitamin E (72.8 ± 3.8%). The ethanolic extract (EE) showed moderate efficacy, with migration rates peaking at 25 µg/mL (60.5 ± 3.6%) but declining at higher concentrations. This suggests that while EE can stimulate fibroblast activity, its efficacy is dose-limited compared to LEC. The blank liposome control showed limited fibroblast migration (43.5 ± 2.6%), indicating that the enhanced migration observed in LEC was due to the active extract encapsulated in the liposomes (Figure 3B).

### 4.5 Wound closure in the excision model

**Figure 4:**
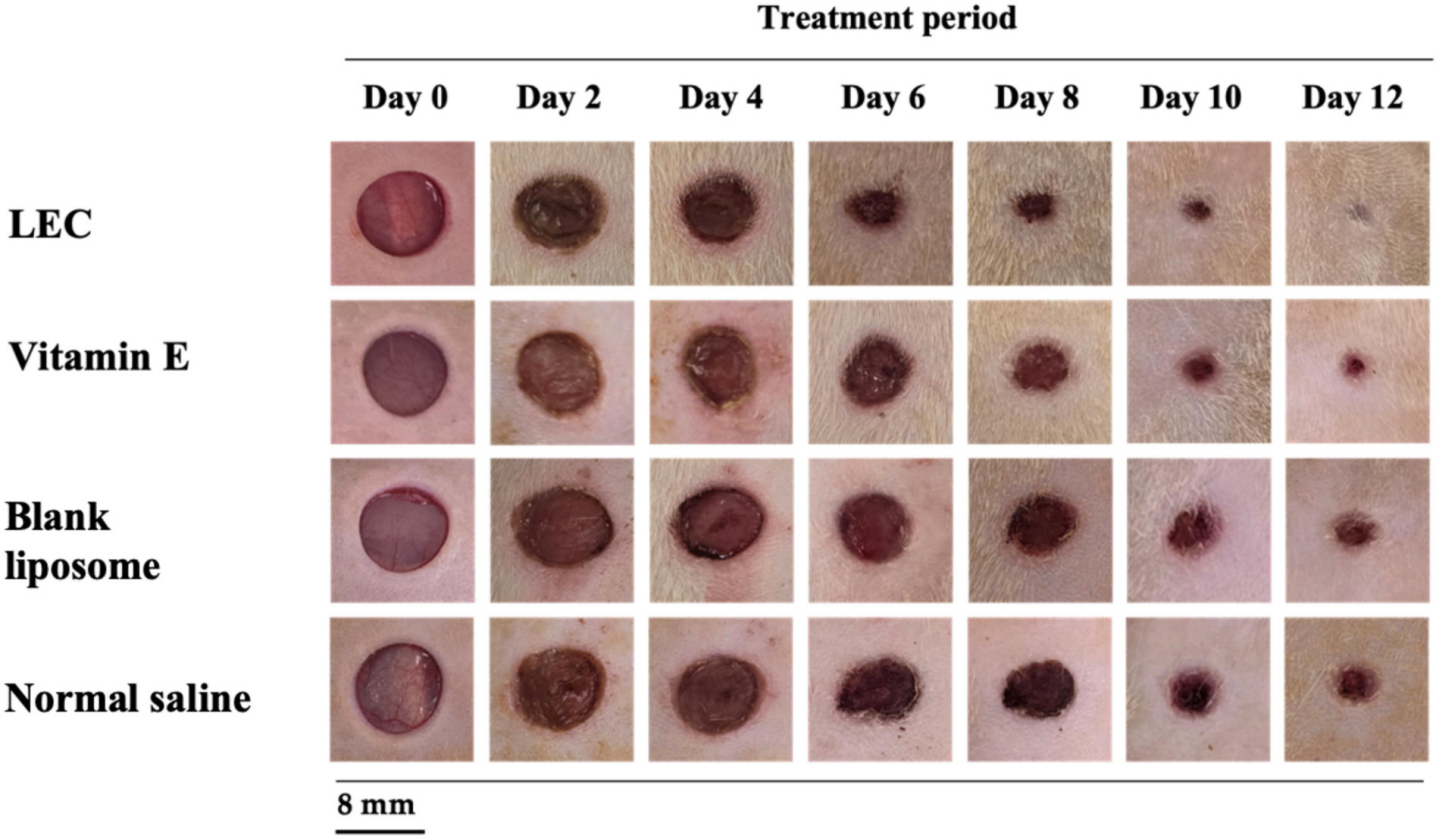
Representative images of wound healing progression in the excision wound model within 12 days. Wounds treated with liposomal-encapsulated *C. asiatica* extract (LEC) show accelerated closure and early tissue regeneration. Wounds treated with vitamin E exhibit progressive healing with well-organized granulation tissue. Blank liposome group shows moderate wound contraction with delayed re-epithelialization. Normal saline group demonstrates the slowest wound healing, with persistent wound gaps and prolonged inflammation.

In the excision wound model, treatment with the liposome-encapsulated *C. asiatica* extract (LEC) gel exhibited a pronounced wound-healing effect compared with the control groups. By Day-2 post-treatment, animals receiving LEC showed a clear reduction in wound area, with the degree of wound closure closely approaching that of the vitamin E-treated group by Day-4. In contrast, wounds in the blank liposome and normal saline control exhibited minimal contraction during the same period (Figure 4).

Quantitative analysis of wound diameter and area confirmed that the LEC-treated group demonstrated a significantly higher percentage of wound contraction than the normal saline group throughout the 12-day observation period (*p* < 0.05; Table 1 and Supplementary Table S1). The rate of wound closure in the LEC group was comparable to that of the vitamin E-treated standard group from Day-4 onward, indicating that the liposomal formulation achieved early and sustained wound-healing efficacy. By Day-8, wounds in the control groups remained noticeably larger, whereas those treated with LEC or vitamin E had nearly complete epithelial coverage. These findings collectively suggest that topical application of LEC significantly accelerates wound contraction and promotes faster tissue regeneration, comparable to standard vitamin E treatment.

**Table 1:** Percentage (%) of wound closure in different groups of animals.

Treatment	Day 2	Day 4	Day 6	Day 8	Day 10	Day 12
LEC	37.0 ± 3.9^a^	53.2 ± 6.3^a^	69.5 ± 6.9^a^	84.1 ± 5.3^a^	94.8 ± 2.2^a^	99.9 ± 0.1^a^
Vitamin E	20.5 ± 2.5^b^	39.3 ± 6.4^b^	50.5 ± 4.2^b^	69.5 ± 3.4^b^	82.0 ± 3.6^b^	90.8 ± 1.6^b^
Blank liposome	16.9 ± 1.3^c^	27.2 ± 3.1^c^	34.7 ± 7.2^c^	50.5 ± 7.6^c^	65.7 ± 9.9^c^	76.9 ± 7.5^c^
Normal saline	12.2 ± 4.7^c^	25.5 ± 2.7^c^	33.8 ± 4.7^c^	42.8 ± 4.8^c^	57.4 ± 3.9^d^	73.7 ± 2.8^c^

Values are expressed as mean ± SD (*n* = 5 rats per group). Two full-thickness excision wounds were created per rat, and wound area measurements from the two wounds were averaged for each animal prior to analysis. Statistical analysis was performed using two-way ANOVA followed by Tukey's multiple-comparison test. Different letters indicate statistically significant differences among treatment groups on the same day (*p* < 0.05).

**Figure 5:**
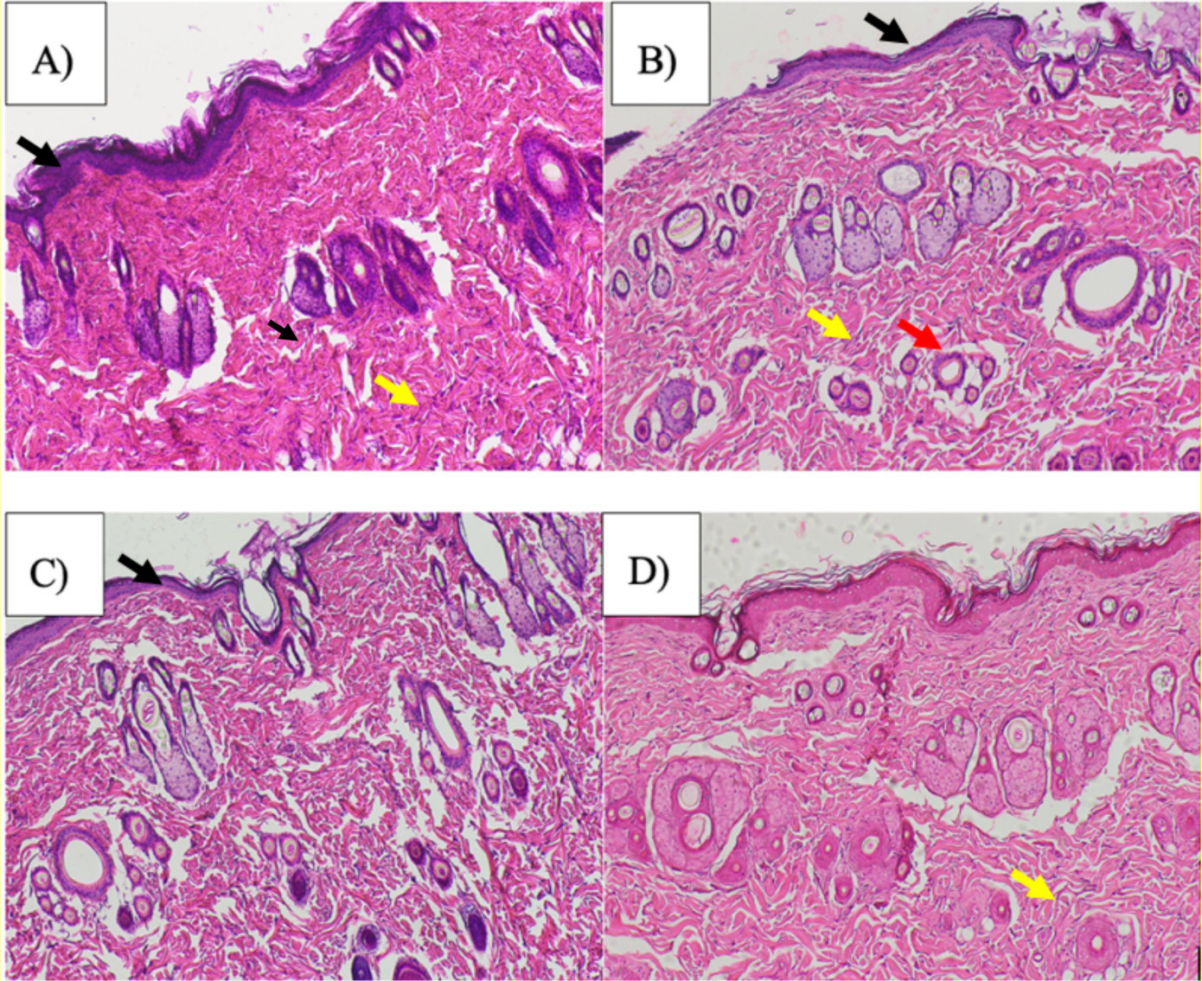
Representative hematoxylin and eosin (H&E)–stained sections of excision wound tissues on Day 12. (**A**) Liposome-encapsulated *C.asiatica* extract (LEC)–treated wounds, (**B**) vitamin E–treated wounds, (**C**) blank liposome control group, and (**D**) normal saline group. Black arrows indicate re-epithelialization and restoration of epidermal continuity, yellow arrows indicate fibroblast proliferation and granulation tissue formation, and red arrows indicate neovascularization. The LEC-treated group demonstrates complete re-epithelialization, dense fibroblast proliferation, and enhanced neovascularization compared with control groups. Scale bar = 400 µM.

Histological examination of excised wound tissues on Day-12 revealed clear treatment-dependent differences in the progression of wound healing (Figures 5, 7B). Wounds treated with liposome-encapsulated *C. asiatica* extract (LEC) exhibited the most advanced histological features of tissue repair. The epidermis was completely re-epithelialized, forming a continuous and well-organized epithelial layer, which was reflected by the highest re-epithelialization score (2.6 ± 0.5). The underlying dermis showed dense fibroblast-rich granulation tissue and well-developed collagen architecture, accompanied by prominent neovascularization, indicating active tissue remodeling and angiogenesis (Figure 5A).

In the vitamin E treated group, wound healing was moderately advanced. The epidermis was largely continuous, although less uniform than in the LEC group, corresponding to an intermediate re-epithelialization score (1.8 ± 0.8). The dermis contained numerous fibroblasts and newly formed blood vessels, with moderately dense collagen deposition, suggesting ongoing granulation tissue formation and angiogenesis (Figure 5B). Semi-quantitative analysis confirmed intermediate fibroblast/granulation and neovascularization scores (1.6 ± 0.5 and 2.0 ± 0.7, respectively). In contrast, the blank liposome and normal saline groups exhibited delayed and less organized wound repair. These groups showed incomplete and fragmented epidermal coverage, sparse fibroblast proliferation, and limited collagen deposition, accompanied by reduced neovascularization (Figures 5C,D). Correspondingly, the re-epithelialization, fibroblast/granulation, and neovascularization scores were significantly lower in these control groups compared with the LEC-treated wounds (Figure 7B). Overall, the semi-quantitative histological scoring corroborates the qualitative microscopic observations and macroscopic wound closure data, demonstrating that liposomal encapsulation of *C. asiatica* extract significantly enhances epidermal regeneration, granulation tissue formation, and angiogenesis, thereby accelerating the wound-healing process.

**Figure 6:**
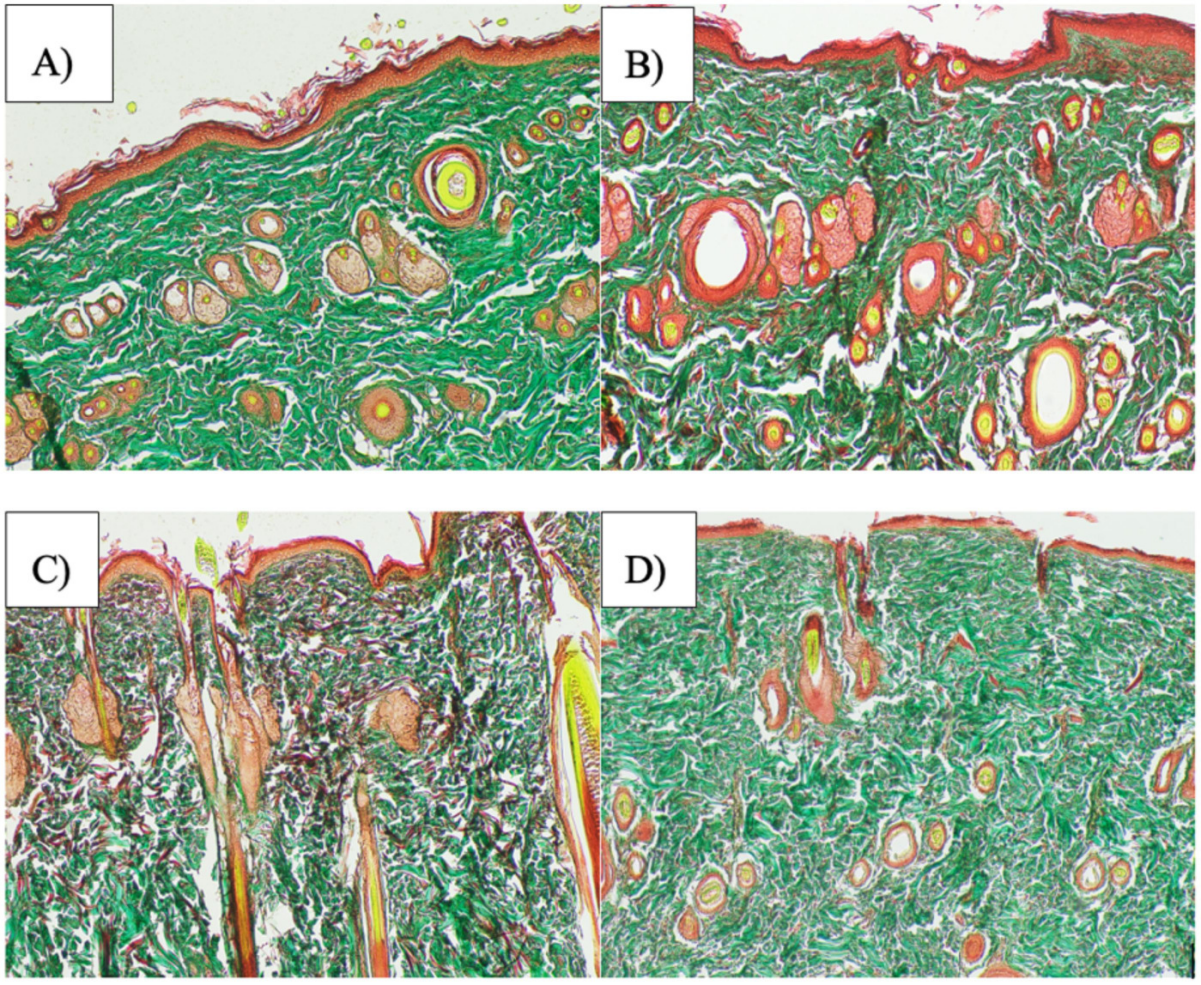
Representative masson's trichrome–stained sections of wound tissues on Day 12: (**A**) LEC treated, (**B**) vitamin E treated, (**C**) blank liposome control, and (**D**) normal saline control groups. Collagen fibers are stained green. Scale ba*r* = 400 µm.

On Day 12 post-treatment, Masson's trichrome staining was performed to evaluate collagen deposition and dermal remodeling in excised wound tissues (Figure 6). Clear treatment-dependent differences in both the extent and organization of collagen fibers were observed among the experimental groups. Wounds treated with liposome encapsulated *C. asiatica* extract (LEC) exhibited the most prominent collagen deposition. The dermis contained densely packed, compact, green-stained collagen fibers that were well aligned and oriented parallel to the epidermal surface, indicating advanced extracellular matrix remodeling. The regenerated epidermis was continuous and well stratified, consistent with near-complete re-epithelialization (Figure 6A).

In the vitamin E treated group, collagen deposition was also evident, with moderately dense and organized collagen fibers distributed throughout the dermis. Although collagen alignment was slightly less compact than that observed in the LEC group, the dermal architecture was largely preserved, with visible adnexal structures such as hair follicles and sebaceous glands. The epidermis appeared intact and stratified, reflecting an ongoing but less advanced remodeling phase compared with LEC treatment (Figure 6B). In contrast, the blank liposome group showed sparse and disorganized collagen fibers that were thin, irregularly oriented, and unevenly distributed within the dermis. The overall tissue architecture appeared disrupted, and epidermal regeneration was incomplete, indicating delayed wound repair (Figure 6C). The normal saline treated group demonstrated the poorest healing response, characterized by minimal collagen deposition with scattered green stained fibers, poor dermal organization, and a thin, fragmented epidermis (Figure 6D).

**Figure 7:**
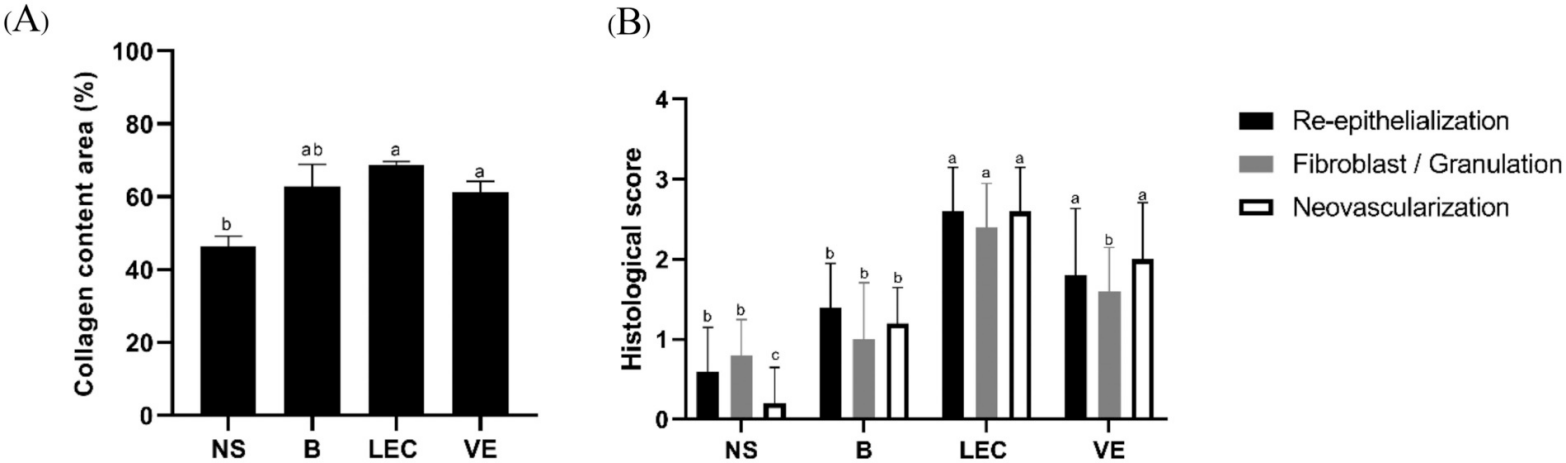
Histological evaluation and collagen deposition in excision wounds on Day 12. (**A**) Collagen content area (%) quantified from Masson's trichrome–stained sections in wounds treated with normal saline (NS), blank liposome (**B**), liposome-encapsulated *C. asiatica* extract (LEC), or vitamin E (VE) (**B**) Semi-quantitative histological scores for re-epithelialization, fibroblast/granulation tissue formation, and neovascularization, assessed using a standardized ordinal scale ranging from 0 (absent) to 3 (marked) Data are presented as mean ± SD (*n* = 5 rats per group). Statistical analysis for collagen area fraction was performed using one-way ANOVA followed by Tukey's multiple-comparison test, while histological scores were analyzed using the Kruskal–Wallis test followed by Dunn's multiple-comparison test. Different letters above bars indicate statistically significant differences among treatment groups within the same parameter (*p* < 0.05).

Quantitative morphometric analysis of Masson's trichrome-stained sections confirmed these observations (Figure 7A). The collagen area fraction was significantly higher in the LEC-treated group compared with the normal saline group (*p* < 0.01). Both the blank liposome and vitamin E groups also showed significantly increased collagen content relative to normal saline (*p* *<* 0.05), although the magnitude of collagen deposition was lower than that observed in the LEC group. Collectively, these findings demonstrate that LEC treatment markedly enhances collagen deposition, dermal organization, and epidermal regeneration, supporting accelerated wound remodeling compared with control and reference treatments.

## 5 Discussion

This study demonstrates that liposome-encapsulated *C. asiatica* ethanolic extract (LEC) enhances anti-inflammatory activity and wound healing responses across complementary *in vitro* and *in vivo* models. By integrating macrophage mediated inflammation assays, fibroblast functional analyses, and an excision wound model, the findings provide evidence that liposomal encapsulation improves the biological performance and cytocompatibility of *C. asiatica* extract compared with the crude ethanolic extract (EE), vitamin E, and control treatments.

Inflammation is a critical early phase of wound repair, however, excessive or prolonged inflammatory responses can delay healing and contribute to chronic wound pathology (24, 26, 27). In the present study, LEC significantly reduced TNF-α and IL-1β production in LPS-stimulated RAW 264.7 macrophages in a dose-dependent manner. Across all tested concentrations, LEC exhibited greater cytokine suppression than EE and the blank liposome control, with effects comparable to or exceeding those of vitamin E. These findings indicate that liposomal encapsulation enhances the anti-inflammatory potential of *C. asiatica*, which is essential for preventing dysregulated inflammatory cascades during early wound repair. The enhanced anti-inflammatory activity of LEC may be attributed to improved solubility, stability, and cellular uptake of key triterpenoid saponins, including asiaticoside and madecassoside (6, 7, 28, 29). Previous studies have shown that lipid-based nanocarriers can increase the bioavailability and therapeutic index of poorly water-soluble herbal compounds (8), supporting the rationale for using liposomes as a delivery platform in this study.

Consistent with the *in vitro* observations, topical application of LEC accelerated wound contraction *in vivo*, with measurable improvements evident from Day-2 and near-complete closure by Day-12. Histological analyses further supported these macroscopic findings, revealing enhanced re-epithelialization, organized collagen deposition, reduced inflammatory cell infiltration, and improved tissue architecture in LEC-treated wounds. Together, these outcomes indicate that LEC facilitates coordinated progression through the inflammatory, proliferative, and remodeling phases of wound healing (1, 10).

Although an extract-only group was not included in the *in vivo* wound-healing model, the intrinsic biological effects of the ethanolic extract were systematically evaluated *in vitro*. At concentrations between 25 and 100 µg/mL, EE reduced fibroblast migratory capacity, whereas LEC significantly enhanced migration while maintaining cytocompatibility. Based on these findings, the *in vivo* study was intentionally designed to focus on the liposomal formulation to avoid potential inhibitory effects of the crude extract on wound repair and to adhere to ethical principles aimed at minimizing unnecessary animal use. This approach allowed a clearer assessment of the therapeutic contribution of liposomal delivery to wound healing outcomes.

The therapeutic effects of LEC are likely mediated through synergistic mechanisms, including attenuation of excessive inflammatory signaling, enhancement of fibroblast-driven tissue regeneration, and improved delivery of bioactive phytochemicals via the liposomal carrier. The relatively early onset of wound contraction observed with LEC may be particularly relevant for clinical contexts requiring rapid tissue repair, such as post-surgical wounds or delayed-healing lesions. Overall, the favorable efficacy and cytocompatibility profile of LEC supports its potential as a topical wound-healing formulation. Compared with conventional antiseptics or corticosteroids, LEC represents a biocompatible, plant derived alternative with multifunctional biological activities. Moreover, liposomal technology offers advantages in formulation stability and controlled delivery, supporting future translational development. Nevertheless, further studies are warranted to validate these findings in chronic wound models (e.g., diabetic or ischemic wounds) and to investigate underlying molecular mechanisms, including NF-κB signaling, oxidative stress modulation, and matrix metalloproteinase activity (30–35). Optimization of formulation stability and dosage form design (e.g., gels, creams, or sprays) will also be essential to facilitate clinical translation.

## 6 Conclusions

The present study demonstrated that liposome-encapsulated *C. asiatica* ethanolic extract (LEC) effectively enhanced wound healing through improved fibroblast proliferation, migration, and tissue regeneration in both *in vitro* and *in vivo* models. The liposomal formulation maintained high cell viability and significantly accelerated fibroblast migration at concentrations between 12.5 and 50 µg/mL, leading to faster wound closure and enhanced re-epithelialization in the excision wound model. These effects are likely attributed to improved stability, sustained release, and enhanced bioavailability of the extract within the liposomal system. Altogether, the findings support the potential of LEC as a biocompatible and efficacious topical formulation for promoting wound repair. Further investigations should explore its molecular mechanisms of action, long-term safety, and anti-inflammatory potential to advance its development as a natural therapeutic agent for wound management.

## Data Availability

The original contributions presented in the study are included in the article/Supplementary Material, further inquiries can be directed to the corresponding author/s.
