# Supplementary material for: Evaluation of liposome-encapsulated Centella asiatica ethanolic extract for enhanced in vitro and in vivo wound healing
Source: Front Med Technol. 2026 Jan 23;8:1740835. doi: 10.3389/fmedt.2026.1740835 (PMC12876173; doi:10.3389/fmedt.2026.1740835)
Supplement: Supplementary file 1 [file Datasheet1.pdf]

Supplementary Table S1 Effect of treatments on wound area (mm^2).

Treatment	Day-0	Day-2	Day-4	Day-6	Day-8	Day-10	Day-12
**LEC**	49.76 ± 0.72	31.21 ± 1.84 [a]	23.26 ± 2.94 [a]	15.12 ± 3.29 [a]	7.91 ± 2.61 [a]	2.58 ± 1.10 [a]	0.03 ± 0.04 [a]
**Vitamin E**	49.72 ± 0.73	40.19 ± 1.33 [b]	30.69 ± 3.13 [b]	25.02 ± 1.95 [c]	15.40 ± 1.77 [c]	9.06 ± 1.71 [c]	4.65 ± 0.85 [a]
**Blank control**	49.82 ± 0.62	41.38 ± 0.56 [d]	36.24 ± 1.32 [e]	32.46 ± 1.19 [e]	25.23 ± 2.09 [a]	17.24 ± 1.98 [a]	11.73 ± 2.06 [a]
**Normal saline**	49.98 ± 0.36	44.21 ± 2.23 [a]	37.53 ± 1.37 [e]	33.34 ± 2.38 [e]	28.83 ± 2.44 [a]	21.48 ± 2.00 [a]	13.26 ± 1.43 [a]

[a] Significant difference compared to all other groups ($p < 0.05$)
[b] Significant difference compared to LEC and normal saline group ($p < 0.05$)
[c] Significant difference compared to LEC, blank control and normal saline group ($p < 0.05$)
[d] Significant difference compared to LEC and normal saline group ($p < 0.05$)
[e] Significant difference compared to LEC, vitamin E ($p < 0.05$)
